# ﻿Uncovering the rich amphibian fauna of two semideciduous forest fragments in southwestern Bahia, Brazil

**DOI:** 10.3897/zookeys.1217.119844

**Published:** 2024-11-04

**Authors:** Carlos Augusto Souza-Costa, Mirco Solé, Caio Vinicius de Mira-Mendes, Antônio Jorge Suzart Argôlo, Iuri Ribeiro Dias

**Affiliations:** 1 Graduate Program in Zoology, Universidade Estadual de Santa Cruz, Rodovia Jorge Amado, km 16, 45662-900, Ilhéus, Bahia, Brazil; 2 Museum Koenig Bonn (ZFMK), Leibniz Institute for the Analysis of Biodiversity Change, Adenauerallee 160, 53113 Bonn, Germany; 3 Graduate Program in Tropical Aquatic Systems, Universidade Estadual de Santa Cruz, Rodovia Jorge Amado, km 16, 45662-900 Ilhéus, Bahia, Brazil; 4 Department of Biology, Universidade Estadual do Maranhão, São Luís, Maranhão 65055-310, Brazil

**Keywords:** Anura, biodiversity, inventory, species distribution, species richness, transitional forest

## Abstract

Fauna inventories reduce biodiversity knowledge gaps by providing comprehensive data on species distribution, richness, and abundance. Furthermore, they identify undocumented species and enhance understanding of ecosystem dynamics and conservation needs. The richness and abundance of amphibian species were studied in two Semideciduous Seasonal Forest areas in the municipalities of Potiraguá (Serra Azul) and Itarantim (Serra do Mandim) in southwestern Bahia, Brazil. Active visual and acoustic surveys were conducted in 24 forest interior transects, two stream transects, and two permanent ponds investigated in the study area. Opportunistic encounters during team movements were also recorded. The richness was 46 amphibian species distributed in 14 families and 26 genera. Approximately half of the species were shared between the two areas, while 11 species were exclusive to Serra Azul and another nine were found only in Serra do Mandim. Cluster analysis for 42 locations in Atlantic Forest, Caatinga, and Cerrado, in a presence/absence matrix with 216 species, revealed that the composition of the amphibians found in Serra do Mandim and Serra Azul is similar to other sampled locations in the northeastern region of Minas Gerais, close to the study site, which are considered transitional between the Atlantic Forest and the Caatinga. Our results demonstrate that the remaining forest fragments in the region, although small and isolated, still sustain a high richness of amphibians with species restricted to the Atlantic Forest and Bahia, such as *Bahiusbilineatus*, *Ololygonstrigilata*, *Aplastodiscusweygoldti* and *Vitreoranaeurygnatha*, and others considered typical of the Caatinga, such as *Leptodactylustroglodytes* and *Physalaemuscicada*. Additionally, we sampled potential new species, filled occurrence gaps, and expanded the geographical range of *Pseudisfusca*.

## ﻿Introduction

Amphibians are considered good environmental indicators due to their permeable skin, exposed eggs and embryos, and generally biphasic life cycle, allowing these organisms to respond to disturbances in both terrestrial and aquatic ecosystems (Wells 2007; Da-Silva et al. 2012; Fonte et al. 2019). Additionally, climate change can affect them, and studies demonstrate that some communities in the Neotropical region are already close to their physiological temperature limits (Duarte et al. 2012; Gutiérrez-Pesquera et al. 2016; Carilo-Filho et al. 2021).

The greatest threat to amphibians and fauna in general is habitat loss and fragmentation, which reduces shelter availability, food supply, isolates populations, and affects their genetic variability (Young et al. 2004; Becker et al. 2007). Amphibians stand as the most endangered class of vertebrates, with 40.7% of their species at risk of extinction (Luedtke et al. 2023). Although the proportion of species classified as Data Deficient (DD) has decreased in the most recent Global Amphibian Assessment (from 22.5% to 11.3%), the high number of species still listed as DD poses a challenge for researchers and hinders effective conservation efforts (Hoffmann et al. 2010; Luedtke et al. 2023).

The Atlantic Forest, originally spanning approximately 1.3 million km^2^, has undergone significant reduction, with estimates suggesting that only between 11.4% and 16% of its original coverage remains (Ribeiro et al. 2009). Nevertheless, the forest remnants still house an exuberant biological diversity, including endemic and threatened species, as well as species with restricted distribution to specific ecosystems (Myers et al. 2000; Haddad et al. 2013; Zappi et al. 2015).

In the southwest of Bahia, the Atlantic Forest is mainly composed of the Seasonal Forest (Deciduous and Semideciduous), which connects to interior forests, such as Caatinga and Cerrado (SOS MATA ATLÂNTICA and INPE 2018). This forest formation has physical and biological characteristics of adjacent regions, allowing faunal elements from other ecosystems to occur in these areas (Willians 1996). Nevertheless, even with the damage caused by human activities, such as pasture creation, logging, and mining (Miles et al. 2006; Silva et al. 2006), few protected areas have been established in the region, such as the REBIO (Biological reserve) Mata Escura and Alto Cariri National Park (ICMBio 2003, 2010) and the RPPN (Private Reserve of Natural Heritage) Mata do Passarinho (ICMBio 2016).

Amphibian surveys in Bahia have revealed significant species richness (e.g., Rojas-Padilla et al. 2020; Protázio et al. 2021; Bastos and Zina 2022), with records of new species (e.g., Vörös et al. 2017; Zucchetti et al. 2023; Santos et al. 2023) and expansions of the geographic distribution (e.g., Dias et al. 2010; Dias et al. 2011; Almeida et al. 2022). Interestingly, a family previously known only from the Amazon was recorded in the state of Bahia through the description of a new species (Caramaschi et al. 2013). Inventories contribute to the knowledge of species richness of a given region, as well as the understanding of the functional structure and population dynamics of amphibians (Droege et al. 1998; Haddad 1998; Camardelli and Napoli 2012). These studies are essential for planning conservation decisions and policies aimed at mitigating anthropogenic effects on species and for the creation of strategic areas for environmental protection (Silvano and Segalla 2005; IUCN 2024). Moreover, they assist in gathering information that enables the reduction of gaps in the distribution and composition of the anurofauna in the country (Rodrigues 2003; Tabarelli and Silva 2003).

Thus, the aim of this study was to conduct an inventory of the amphibians in two remaining Semideciduous Forests in the southwest of Bahia, comparing the amphibian community of these remnants with others from the Atlantic Forest, Caatinga, and Cerrado.

## ﻿Materials and methods

### ﻿Study area

**Figure 1.**
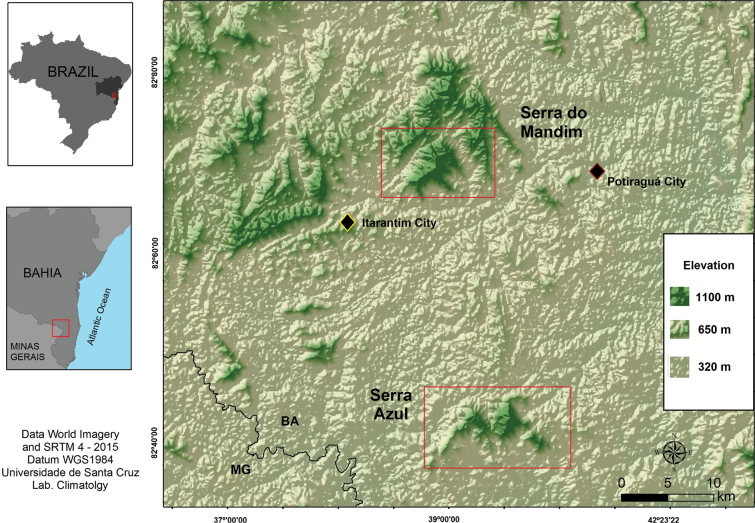
Study areas in the southwest region of Bahia, Brazil. Serra do Mandim belongs to the municipality of Itarantim, Bahia and Serra Azul, one of its portions inserted in the municipality of Potiraguá, Bahia.

**Figure 2.**
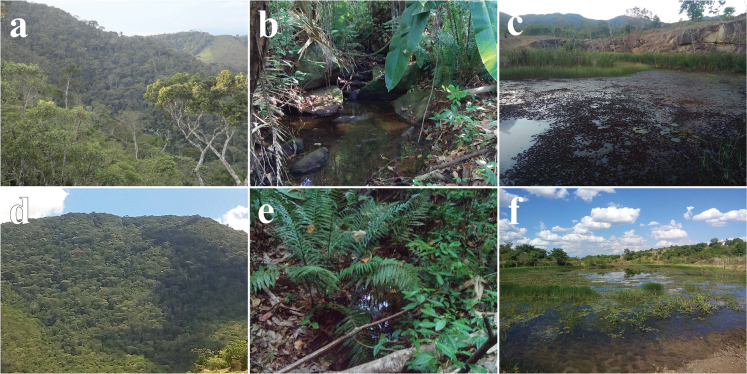
Study areas in the southwest region of Bahia. Fugiama farm in Serra do Mandim (**a**, **b, c**) **a** semideciduous forest fragment **b** stream **c** permanent pond. Serra Azul farm in Serra Azul (**d**, **e, f**) **d** semideciduous forest fragment **e** stream **f** permanent pond.

The study was carried out in two fragments of Atlantic Forest in the Southwest region of Bahia: Fugiama farm (15°37'58"S, 39°59'01"W), with approximately 120 hectares of forest, located in the Serra do Mandim, municipality of Itarantim, and Serra Azul farm (15°52'01"S, 39°55'54"W), with about 160 hectares of forest, located in the Serra Azul, municipality of Potiraguá, both located in the state of Bahia (Figs 1, 2). While the mountains themselves reach up to approximately 1100 m in altitude, the areas accessed during the study were at around 800 m in altitude (Table 1).

**Table 1.** Sampling points of the amphibian survey, coordinates, altitude, and sampling methods in Serra do Mandim, municipality of Itarantim and Serra Azul, municipality of Potiraguá, state of Bahia. TF = Transects in the forest; TS = Transects in the streams; P = permanent ponds.

Locality	Sampling points	Coordinates (Latitude; Longitude)	Altitude (m)	Sampling method
**Serra do Mandim-BA**	01	15°37'39.2"S, 39°58'41.6"W	728 m	TF
	02	15°37'38.9"S, 39°58'39.2"W	704 m	TF
	03	15°37'40.8"S, 39°58'37.5"W	672 m	TF
	04	15°37'47.9"S, 39°58'35.2"W	584 m	TF
	05	15°37'50.4"S, 39°59'02.2"W	758 m	TF
	06	15°37'49.5"S, 39°58'59.9"W	681 m	TF
	07	15°37'47.6"S, 39°58'57.9"W	635 m	TF
	08	15°37'47.8"S, 39°58'50.7"W	560 m	TF
	09	15°37'47.6"S, 39°58'48.9"W	587 m	TF
	10	15°37'47.5"S, 39°58'46.1"W	574 m	TF
	11	15°37'36.9"S, 39°58'43.5"W	755 m	TF
	12	15°37'51.4"S, 39°58'35.9"W	513 m	TF
	25	15°37'54.1"S, 39°58'34.0"W	485 m	TS
	27	15°39'15.4"S, 39°59'00.8"W	250 m	P
**Serra Azul-BA**	13	15°52'21.8"S, 39°54'30.6"W	731 m	TF
	14	15°52'19.7"S, 39°54'27.1"W	800 m	TF
	15	15°52'22.0"S, 39°54'22.7"W	739 m	TF
	16	15°52'24.7"S, 39°54'22.2"W	690 m	TF
	17	15°52'27.7"S, 39°54'22.5"W	672 m	TF
	18	15°52'26.3"S, 39°54'18.6"W	780 m	TF
	19	15°52'28.1"S, 39°54'10.9"W	692 m	TF
	20	15°52'25.9"S, 39°54'29.3"W	761 m	TF
	21	15°52'29.0"S, 39°54'27.7"W	650 m	TF
	22	15°52'34.5"S, 39°54'22.0"W	668 m	TF
	23	15°52'33.2"S, 39°54'25.0"W	652 m	TF
	24	15°52'29.7"S, 39°54'24.9"W	648 m	TF
	28	15°52'33.7"S, 39°54'24.2"W	658 m	TS
	26	15°51'52.5"S, 39°53'26.1"W	256 m	P

The region borders the state of Minas Gerais and is located between two neighboring basins: the Pardo River basin and the Jequitinhonha River basin (IBGE 1997). Both areas are inserted in the Phytogeographic Domain of the Semideciduous Forest, characterized by the presence of climate type Am, with one to three dry months (Köppen 1936) and are ~ 28 km apart from each other. The climatic characteristics of the region encompass humid and sub-humid climates, defined by rainfall between 800 and 1100 mm and thermal averages from 23.5 to 25 °C (IBGE 1997).

The vegetation of the study area is characterized as Semideciduous Forest (SOS MATA ATLÂNTICA and INPE 2018). It occurs latitudinally parallel to the formation zone of the Ombrophilous Forest (coastal), at a distance ranging from 20 km to 140 km from the coast (Mori and Silva 1980; Argôlo 2004).

### ﻿Sample design

A total of six field campaigns were carried out between December 2014 and March 2016 in Serra do Mandim and Serra Azul. Each expedition lasted approximately seven days, with approximately three or four days dedicated to active search in each mountain range.

A total of 24 transects were used between altitudes of 500–800 meters with a length of 50 meters and a width of 5 meters, spaced 100 meters apart in a forested area, two transects of 120 meters in streams, and two ponds were selected in a lowland area (Table 1). Twelve transects were sampled in forest, one in a stream, and a permanent pond in each of the areas (Figs 1, 2).

The transects in the forest were surveyed for 40 min, totaling 96 h. The streams in the forest were sampled for 90 min each, totaling 18 h. The permanent ponds were sampled for 30 min each, totaling six h. The sampling was carried out by two researchers. The total sampling effort was 44 days of sampling and 240 h/person. Amphibians were sampled using visual and acoustic active search methods (Heyer et al. 1994; Crump and Scott 1994) and by opportunistic encounters (i.e., along roads or trails outside the transects).

All animals were collected with a license from the Brazilian Institute of Environment and Renewable Natural Resources-IBAMA and/or ICMBio (No. 13708), together with the permission of the administrators of the local farms. Some specimens were euthanized through the administration of a lethal dose of 5% xylocaine to the ventral region, followed by fixation in 10% formaldehyde and preservation in 70% alcohol. All specimens were deposited in the Zoology Museum of the State University of Santa Cruz - MZUESC (Appendix 1).

### ﻿Data analysis

For each species, the total number of individuals observed visually and acoustically was recorded in each sampling unit and environment. To evaluate sample sufficiency, rarefaction curves were constructed based on individuals. Four curves were made for each study area: a general curve considering all individuals sampled in the three standardized methodologies (transect in the forest, transect in the streams and ponds), and three others using individuals collected in each separate methodology. To extrapolate species richness, four non-parametric estimators were used: Chao 2, Jackknife 1, Jackknife 2, and Bootstrap (Magurran 1988; Colwell and Coddington 1994; Toti et al. 2000; Gotelli and Colwell 2001). The analysis was performed using presence/absence data for species during each sampling campaign, with 1000 randomizations.

The species richness recorded in the study area was compared to 42 locations, mostly in the northeast region of Brazil and a smaller number in the northeast region of Minas Gerais, sampled in the Atlantic Forest, Caatinga, and Cerrado (see Table 3). The information extracted from these locations was used to create a binary matrix of presence/absence with 216 amphibian species. The analysis of similarity, considering the specific composition of these areas, was performed using the Jaccard index to calculate dissimilarity and the UPMGA (Unweighted Pair Group Method with Arithmetic) linkage method. Species found in inventories with taxonomic doubts (sp., gr., and aff.) were excluded from the analyses. Subsequently, an ANOSIM (Analysis of Similarities) test was performed considering 9999 permutations, to determine whether the composition of samples recovered in the similarity analysis differs significantly among the groups (Atlantic Forest, Caatinga, and Cerrado). All analyses were conducted using the PAST 4.12 software (Hammer et al. 2001)

The species were identified based on their original descriptions, redescriptions, or recent taxonomic revisions. In addition to the original descriptions, references consulted for species identification are provided in Table 2. Additionally, the collected material was compared with specimens identified at MZUESC. For nomenclature, we followed Frost (2023), who also maintains an updated database containing all available synonyms for amphibians worldwide. Regarding *Adelophryne* spp. we follow Lourenço-De-Moraes et al. (2018). The conservation status of the species was classified according to IUCN (2024). Furthermore, we verified which species are endemic of the Atlantic Forest based on Rossa-Feres et al. (2017).

**Table 2.** Species of amphibians from Serra do Mandim and Serra Azul, southwest Bahia, Brazil. Legend: **SM** = Sampling Method (**OE** = Oportunistic encounters, **TF** = Transect in the forest, **P** = Ponds, **TS** = Transect in the streams). **HAB** = Habitat (**LL** = Leaf litter or understory, **B** = Bromeliads or epiphytes, **S** = Streams, **F** = Forest, **P** = Ponds or open area). **N** = Number of registered specimens. Additional ID references = Additional references consulted to identify species. # = only acoustic record; † = species only found in the inner forests; * endemic to the Atlantic Rainforest.

Order/Family/Species	Serra do Mandim			Serra Azul			Additional ID references
	SM	HAB	N	SM	HAB	N	
**ANURA**							
**Brachycephalidae**							
*Ischnocnemaverrucosa* (Reinhardt & Lütken, 1862)^†*^	TF	LL	02	TF	LL	06	Lynch 1972; Canedo et al 2010; Araújo et al. 2023
*Ischnocnema* sp. (gr.parva) ^†^	-	-	-	TF	LL	01	Heyer et al. 1990; Silva-Soares et al. 2021
**Bufonidae**							
*Dendrophryniscusproboscideus* (Boulenger, 1882)^†*^	OE,TF	LL	08	-	-	-	Izecksohn 1976; Caramaschi 2012
*Rhinellacrucifer* (Wied-Neuwied, 1821)*	TF,TS	P,LL	07	OE	P,LL	01	Baldissera et al. 2004; Oliveira et al. 2014
*Rhinelladiptycha* (Cope, 1862)	P	P,LL	02	P	P,LL	09	Stevaux 2002; Lavilla and Brusquetti 2018
*Rhinellagranulosa* (Spix, 1824)	-	-	-	OE,TF,P	P,LL	61	Narvaes and Rodrigues 2009; São Pedro et al. 2011
**Craugastoridae**							
*Haddadusbinotatus* (Spix, 1824)^†*^	TF	LL	51	OE,TF,TS	LL	145	Heyer et al. 1990; Dias et al. 2012
**Centrolenidae**							
*Vitreoranaeurygnatha* (A. Lutz, 1925)^†*^	TS	S	15	-	-	-	Heyer et al. 1990; Zucchetti et al. 2023
**Cycloramphidae**							
*Thoropamiliaris* (Spix, 1824)^†*^	OE,TF,TS	S,LL	10	OE,TS	S,LL	05	Feio et al. 2006a
**Eleutherodactylidae**							
*Adelophryne* sp.8^†^	TF	LL	08	OE,TF	LL	27	Lourenço-de-Moraes et al. 2018
*Adelophryne* sp.2^†^	TF,TS	LL	13	OE,TF	LL	29	Lourenço-de-Moraes et al. 2018
**Hemiphractidae**							
*Gastrothecapulchra* Caramaschi & Rodrigues, 2007^#†*^	-	-	-	OE	B	01	Duellman 2015
**Hylidae**							
*Aplastodiscusweygoldti* (Cruz & Peixoto, 1987)^†*^	TS	S	06	TS	S	08	Orrico et al. 2006
*Boanacrepitans* (Wied-Neuwied, 1824)	OE,P	P	18	OE,TF,P	P	21	Orrico et al. 2017
*Boanaexastis* (Caramaschi & Rodriguez, 2003)^†*^	TF	F	01	-	-	-	Loebmann et al. 2008
*Boanafaber* (Wied-Neuwied, 1821)*	OE,TF,P	P,F	11	OE,TF	P,F	06	Martins and Haddad 1988; Heyer et al. 1990
*Dendropsophusbranneri* (Cochran, 1948)	OE,P	P	64	OE,P	P	83	Bastos and Pombal 1996; Nunes et al. 2007; Orrico et al. 2021
*Dendropsophuselegans* (Wied-Neuwied, 1824)*	OE,P	P	52	OE,P	P	24	Gomes and Peixoto 1991; Dias et al. 2017a; Pirani et al. 2022
*Dendropsophusoliveirai* (Bokermann, 1963)	OE,P	P	60	OEP	P	107	Santana et al. 2011; Orrico et al. 2021
*Ololygonstrigilata* (Spix, 1824)^†*^	OE	S	01	-	-	-	Pimenta et al. 2007
*Phyllodytesmaculosus* Cruz, Feio & Cardoso, 2007^#†*^	-	-	-	TF	B	01	Dias et al. 2020
*Phyllodytesluteolus* (Wied-Neuwied, 1821)*	OE	B	02	-	-	-	Bokermann 1966a; Blotto et al. 2021
*Pithecopusnordestinus* (Caramaschi, 2006)	OE,P	P	30	P	P	13	Vilaça et al. 2006; Vaz-Silva et al. 2020
*Pseudisfusca* Garman, 1883*	-	-	-	P	P	04	Caramaschi and Cruz 1998; Garda et al. 2010
*Scinaxeurydice* (Bokermann, 1968)*	OE,TF	P,F	03	TF	P,F	01	Magrini et al. 2011; Novaes-e-Fagundes et al. 2016; Menezes et al. 2016
*Scinaxpachycrus* (Miranda-Ribeiro, 1937a)	-	-	-	P	P	07	Carneiro et al. 2004; Novaes-e-Fagundes et al. 2016
*Scinaxx-signatus* (Spix, 1824)	OE,P	P	07	OE	P	01	Araujo-Vieira et al. 2020a; Novaes-e-Fagundes et al. 2021
*Sphaenorhynchusprasinus* Bokermann, 1973*	OE,P	P	16	OE,P	P	29	Araujo-Vieira et al. 2020b
*Trachycephalusnigromaculatus* von Tschudi, 1838*	OE	P,F	01	OE	P,F	04	Bokermann 1966b
**Leptodactylidae**							
*Leptodactylusfuscus* (Schneider, 1799)	P	P	58	OE,P	P	28	Heyer 1978; Heyer et al. 1990; De-Sá et al. 2014
*Leptodactyluslatrans* (Steffen, 1815)	OE,TS,P	P,S	07	P	P,S	06	Magalhães et al. 2022
*Leptodactylusmacrosternum* Miranda-Ribeiro, 1926	TS	P,S	01	P	P,S	01	Magalhães et al. 2022
Leptodactyluscf.mystaceus (Spix, 1824)	OE	S	01	-	-	-	Toledo et al. 2005; De-Sá et al. 2014; Cassini et al. 2013
*Leptodactylusmystacinus* (Burmeister, 1861)	P	P	03	P	P	04	Abrunhosa et al. 2001; De-Sá et al. 2014; Cassini et al. 2013
*Leptodactylustroglodytes* Lutz, 1926	-	-	-	OE,P	P	04	De-Sá et al. 2014
*Leptodactylusviridis* Jim & Spirandeli-Cruz, 1973*	P	P	07	-	-	-	Magalhães et al. 2022
*Physalaemuscicada* Bokermann, 1966c	-	-	-	OE,P	P	05	Nascimento et al. 2005; Hepp and Pombal 2020
Physalaemuscf.erikae Cruz & Pimenta, 2004*	OE,P	P	18	OE,P	P	19	Nascimento et al. 2005; Hepp and Pombal 2020
*Physalaemuskroyeri* (Reinhardt & Lütken, 1862)	-	-	-	OE,P	P	06	Nascimento et al. 2005; Hepp and Pombal 2020; Braga et al. 2024
**Microhylidae**							
*Dermatonotusmuelleri* (Boettger, 1885)	-	-	-	OE	P	02	Vaz-Silva et al. 2020; Dubeux et al. 2021
**Odontophrynidae**							
*Proceratophrysschirchi* (Miranda-Ribeiro, 1937b)^†*^	OE,TF,TS	S,LL	59	OE,TS	S,LL	09	Izecksohn and Peixoto 1980; Sichieri et al. 2021
**Pipidae**							
*Pipacarvalhoi* (Miranda-Ribeiro, 1937a)	P	P	04	-	-	-	Lima et al. 2020
**Strabomantidae**							
*Bahiusbilineatus* (Bokermann, 1975) ^†*^	OE,TF	LL	17	-	-	-	Dias et al. 2017b
*Pristimantisvinhai* (Bokermann 1975) ^†*^	OE,TF,TS	LL	276	OE,TF,TS	LL	178	Trevisan et al. 2020
*Pristimantis* sp. (gr.ramagii)^†^	-	-	-	OE,TF	LL	90	Trevisan et al. 2020
**GYMNOPHIONA**							
**Siphonopidae**							
*Siphonopsannulatus* (Mikan 1822)^†^	-	-	-	TF	LL	01	Maciel and Hoogmoed 2011

## ﻿Results

**Figure 3.**
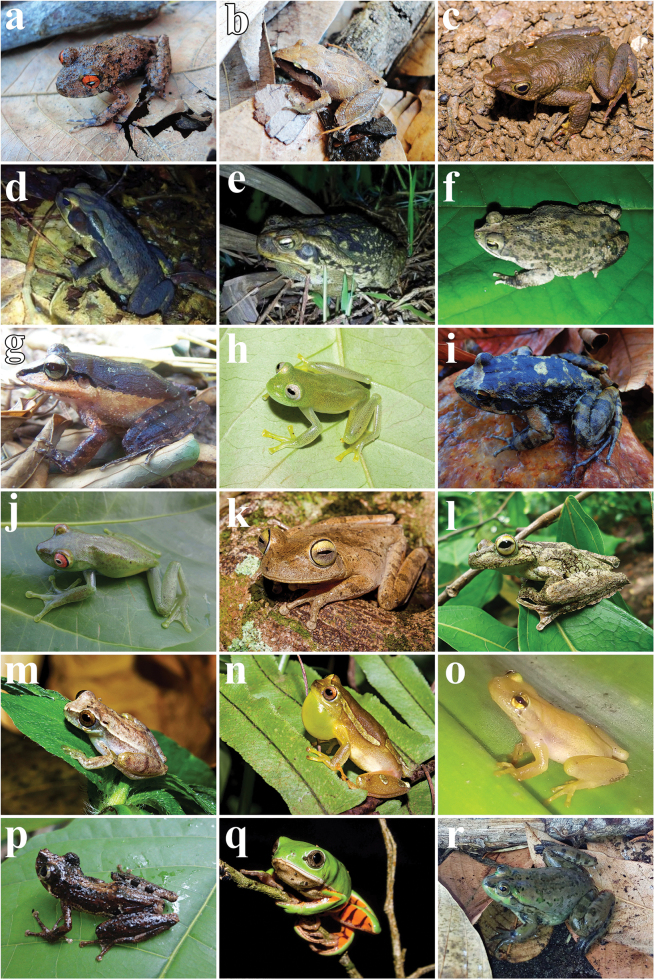
Amphibians registered in Serra do Mandim and Serra Azul in southwestern Bahia, Brazil. **a***Ischnocnemaverrucosa* (MZUESC 15874) **b***Ischnocnema* sp. (gr.parva – MZUESC 15896) **c***Dendrophryniscusproboscideus* (MZUESC 14688) **d***Rhinellacrucifer* (MZUESC 15148) **e***R.diptycha* (MZUESC 15503) **f***R.granulosa* (MZUESC 15055) **g***Haddadusbinotatus* (MZUESC 15646) **h***Vitreoranaeurygnatha* (MZUESC 14691) **i***Thoropamiliaris* (MZUESC 15782) **j***Aplastodiscusweygoldti* (MZUESC 15787) **k***Boanacrepitans* (MZUESC 14675) **l***B.exastis* (MZUESC 15108) **m***Dendropsophusbranneri* (MZUESC 14683) **n***D.elegans* (MZUESC 14679) **o***Phyllodytesluteolus* (MZUESC 17501) **p***Ololygonstrigilata* (MZUESC 15001) **q***Pithecopusnordestinus* (MZUESC 14682) **r***Pseudisfusca* (MZUESC 16528).

**Figure 4.**
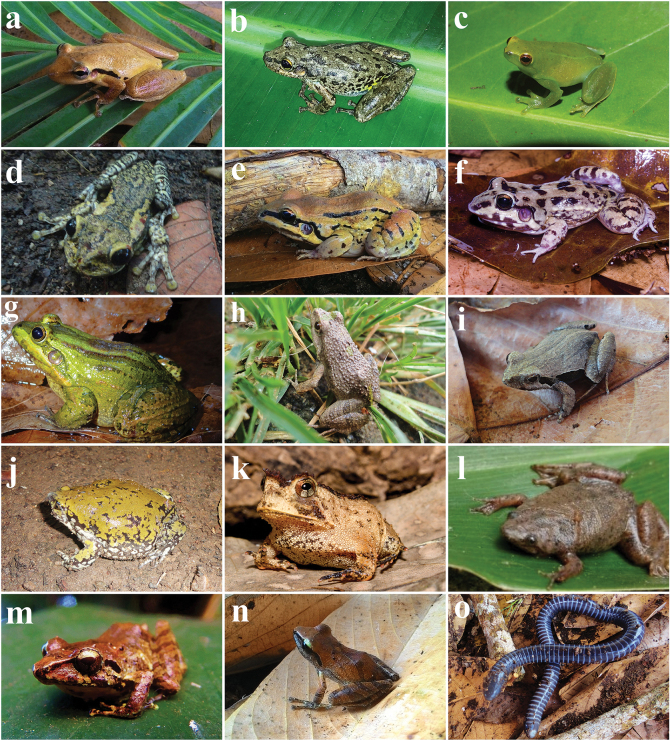
Amphibians registered in Serra do Mandim and Serra Azul in southwestern Bahia, Brazil **a***Scinaxpachycrus* (MZUESC 16525) **b***S.x-signatus* (MZUESC 17503) **c***Sphaenorhynchusprasinus***d***Trachycephalusnigromaculatus* (MZUESC 15064) **e***Leptodactylusmystacinus* (MZUESC 16529) **f***L.troglodytes* (MZUESC 15003) **g***L.viridis* (MZUESC 15848) **h**Physalaemuscf.erikae (MZUESC 15878) **i***P.kroyeri* (MZUESC 15784) **j***Dermatonotusmuelleri* (MZUESC 15070) **k***Proceratophrysschirchi* (MZUESC 14689) **l***Pipacarvalhoi***m***Pristimantisvinhai* (MZUESC 15642) **n***P.* sp (gr.ramagii – MZUESC 16523) **o***Siphonopsannulatus* (MZUESC 15900).

A total of 1785 individuals across 46 amphibian species were recorded, encompassing one species of Gymnophiona (*Siphonopsannulatus*) and 45 anuran species across 14 families (Table 2, Figs 3, 4). The majority of the identified species (*n* = 24; 53%) are endemic to the Atlantic Rainforest (Rossa-Feres et al. 2017). The Hylidae family was the most representative with 36.9% (*n* = 17), followed by the Leptodactylidae family, with 21.7% (*n* = 10) of the amphibians found. The richness identified stands among the highest ever recorded for the northeastern region of Brazil (Table 3). Furthermore, all recognized species identified are listed as Least Concern (LC) on the IUCN Red List (IUCN 2024).

**Table 3.** The number of amphibian species (S), study duration (SD in months), and region (R) type of different study sites in northeastern Brazil, including the northeastern portion of Minas Gerais. Localities listed as RPPN are Private Natural Heritage Reserves, those labelled as APA are Environmental Protection Areas, EE represents Ecological Stations, and PN denotes National Parks. Region abbreviations include Atlantic Forest (AF), Caatinga (CA), and Cerrado (CE).

Localities, states of Brazil	S	SD	R	Source
RPPN Serra Bonita, BA	80	16	AF	Dias et al. 2014b
RE Michelin, BA	69	30	AF	Camurugi et al. 2010; Mira-Mendes et al. 2018
APA Lagoa Encantada and River Almada, BA	59	01	AF	Dias et al. 2014a
Serra da Jibóia, BA	55	~ 20 years	AF	Juncá, 2006; Freitas et al. 2018
Serra do Timbó, BA	55	12	AF	Freitas et al. 2019
PN Serra das Lontras, BA	49	07	AF	Rojas-Padilla et al. 2020
PN Grande Sertão Veredas, BA/GO/MG	47	~ 11 years	CE	Brandão et al. 2020
**Serra Mandim and Serra Azul, BA**	**46**	**08**	**AF**	**This study**
Middle Jequitinhonha River, MG	46	29	CA/CE	Feio and Caramaschi 1995
Chapada Diamantina, BA	44	06	CA	Juncá 2005
RPPN Frei Caneca, PE	42	12	AF	Santos and Santos 2011
Complex Limoeiro, MG	39	03	AF	Feio et al. 2006b
RPPN Estação Veracel, BA	39	01	AF	Silvano and Pimenta 2003
Complex Nossa Senhora Fatima, MG	38	02	AF	Feio et al. 2006b
Planalto de Ibiapaba, CE	38	24	AF/CA	Loebmann and Haddad 2010
Tocantins River Basin, MA/TO	38	06	CE	Brasileiro et al. 2008
Complex Cariri, BA/MG	36	03	AF	Feio et al. 2006b
Morro do Mara, BA	36	15	AF/CA	Bastos and Zina 2022
EE Serra Geral do Tocantins, TO	36	02	CE	Valdujo et al. 2011
Guaratinga, BA	34	01	AF	Silvano and Pimenta 2003
Macaíba, RN	34	14	AF/CA	Magalhães et al. 2013
Conde, BA	33	04	AF	Gondim-Silva et al. 2016
São Desiderio, BA	32	02	CE	Valdujo et al. 2009
Camamu, BA	32	01	AF	Silvano and Pimenta 2003
Serra do Brejo Novo, BA	32	19	AF/CA	Lantyer-Silva et al. 2013
PN Chapada Diamantina, BA	31	01	CA	Magalhães et al. 2015
Cruz das Almas, BA	31	39	AF	Protázio et al. 2021
Complex Bandeira, BA/MG	30	02	AF	Feio et al. 2006b
Complex Santana, MG	28	02	AF	Feio et al. 2006b
Complex Mumbuca, MG	27	02	AF	Feio et al. 2006b
RPPN Sapiranga, BA	25	05	AF	Juncá 2006
PN Descobrimento, BA	25	01	AF	Silvano and Pimenta 2003
Itapebi, BA	24	01	AF	Silvano and Pimenta 2003
EE Raso da Catarina, BA	21	13	CA	Garda et al. 2013
PN Catimbau, PE	21	01	CA	Pedrosa et al. 2014
Curimataú, PB	21	02	CA	Arzabe et al. 2005
Middle Jaguaribe River, CE	19	01	CA	Santana et al. 2015
RPPNs in Betânia and Floresta, PE	19	07	CA	Borges-Nojosa and Santos 2005
Jatobá, PE	18	04	CA	Silva et al. 2011
RPPN Serra das Almas, CE	18	02	CA	Borges-Nojosa and Cascon 2005
Paulo Afonso, BA	17	20	CA	Protázio et al. 2010
Cariri Paraibano, PB	16	23	CA	Vieira et al. 2007

The amphibian richness recorded was similar in the two analyzed areas: Serra do Mandim (*n* = 34) and Serra Azul (*n* = 37), with 25 species shared between the areas and the rest divided, with nine exclusive to Serra do Mandim and 11 to Serra Azul (Tables 2, 4). Among the total species recorded in each area, four species (*Ololygonstrigilata*, *Phyllodytesluteolus*, *Trachycephalusnigromaculatus*, and Leptodactyluscf.mystaceus) were sampled exclusively through opportunistic encounters in Serra do Mandim, while another five (*Rhinellacrucifer*, *Gastrothecapulchra*, *Scinaxx-signatus*, *Trachycephalusnigromaculatus* and *Dermatonotusmuelleri*) also corresponded to opportunistic encounters in Serra Azul.

**Table 4.** Number of species and abundance found in the study area through standardized methodologies and extrapolated richness using richness estimators. **TF**: Transect in the forest; **TS**: Transect in the streams and **P**: Ponds.

Estimators	Serra Azul				Serra do Mandim			
	TF	TS	P	Total	TF	TS	P	Total
**Richness**	13	5	18	32	13	9	15	30
**Chao 2**	15.1±2.7	5.0±0.2	25.8±7.4	39.6±6.3	15.8±3.5	9.8±1.5	15.6±1.2	35.8±6.0
**Jackknife1**	17.2±2.0	5.8±0.8	24.7±4.9	41.2±4.4	17.2±1.5	11.6±1.7	17.5±1.1	35.8±27
**Jackknife2**	18.9	5.9	28.9	45.8	19.4	12.4	17.9	39.4
**Bootstrap**	14.9	5.4	20.8	36.2	14.9	10.2	16.3	32.6
**Abundance**	433	43	392	868	350	106	330	786

In Serra do Mandim most species were found in the monitored pond in the region. Although this was the smallest sampled fragment, some species considered rare and/or with restricted distribution were found only in this location, such as *Dendrophryniscusproboscideus*, and typical stream species such as *Vitreoranaeurygnatha* and *Ololygonstrigilata*.

The anuran richness recorded in Serra Azul was 37 species, and the only species of Gymnophiona recorded in the study was found in this area (Table 2; Fig. 4). Two species were only detected by vocalization, *Phyllodytesmaculosus* and *Gastrothecapulchra*. The first vocalized in bromeliads during forest transect sampling, while the latter was registered vocalizing in the canopy of the forest.

A higher number of individuals was recorded in Serra Azul (*n* = 868) compared to Serra do Mandim (*n* = 786). The most common species during the study were *Pristimantisvinhai* (*n* = 454), *Haddadusbinotatus* (*n* = 296), and *Dendropsophusoliveirai* (*n* = 167). A total of 131 specimens were collected during opportunistic encounters, 78 at Serra Azul and 53 at Serra do Mandim (Table 2).

**Figure 5.**
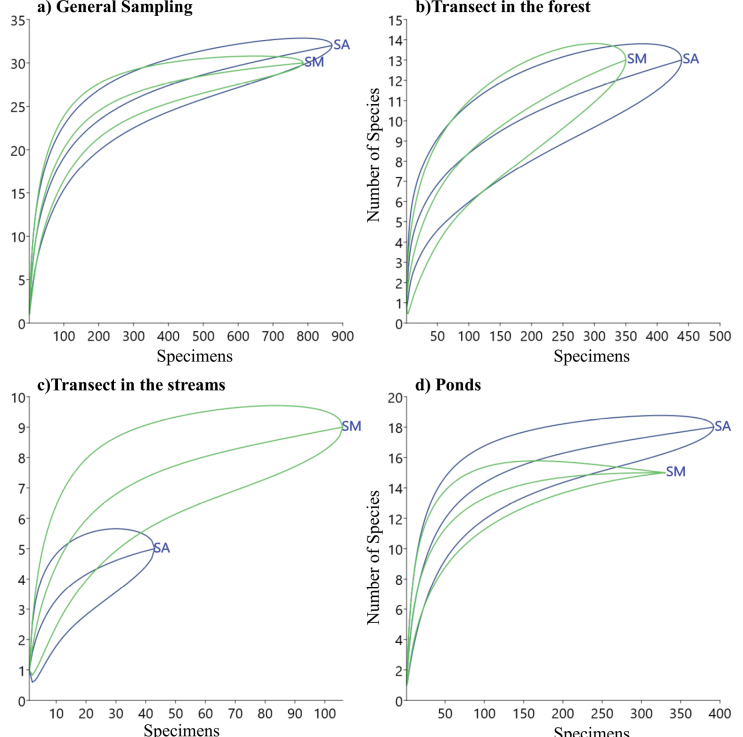
Rarefaction curve based on amphibian individuals for the two fragments of Semi-deciduous Seasonal Forest in Serra do Mandim and Serra Azul in southwest Bahia, Brazil **a** general sampling: Serra do Mandim and Serra Azul (without considering opportunistic records) **b** transect in the forest **c** transect in the streams and **d** ponds. The central line corresponds to the average obtained with 1000 randomizations, and the lines above and below correspond to the 95% confidence interval. The blue line represents Serra Azul (SA), and the green line represents Serra do Mandim (SM).

The overall rarefaction curve obtained for each area showed a tendency towards stabilization but did not reach the asymptote (Fig. 5A). The curves made for the different employed methodologies did not show a stabilization trend, except for the species recorded in ponds (Fig. 5D). The richness estimators suggested the occurrence of between 39–46 species in Serra Azul and between 36–39 species in Serra do Mandim (Table 4). Thus, during field activities, between 78.2–94.4% of the estimated richness for each area was sampled. A summary of the richness estimates for each area and the employed methodologies can be found in Table 4.

**Figure 6.**
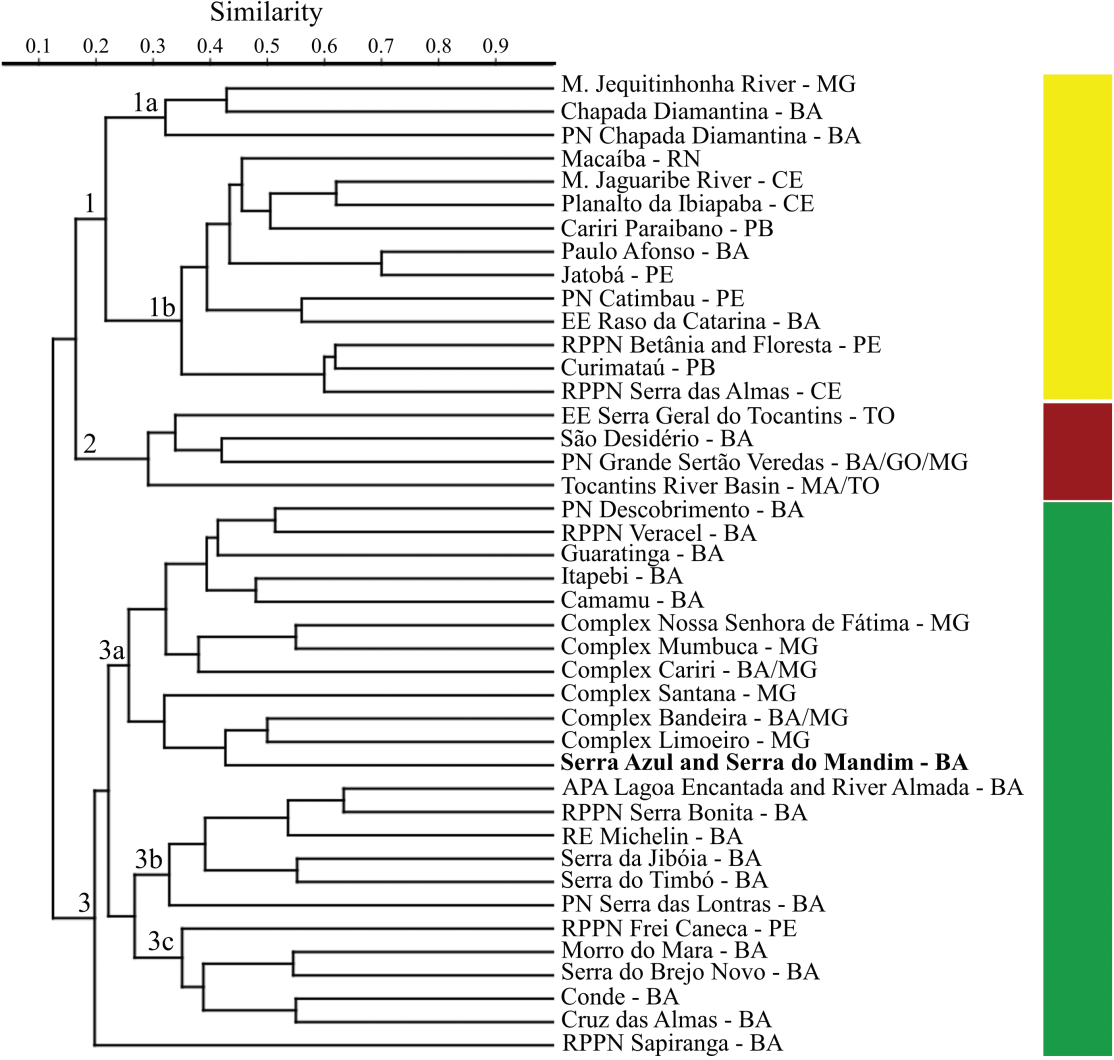
Similarity dendrogram for 42 locations, comparing amphibian composition primarily in the northeast region of Brazil. Jaccard index for dissimilarity calculation and UPGMA (Unweighted Pair Group Method with Arithmetic) clustering method (cophenetic correlation coefficient = 0.798). Green: locations sampled in the Atlantic Forest; Red: locations sampled in the Cerrado; Yellow: locations sampled in the Caatinga.

Cluster analysis yielded three main groups, which are mainly based on different regions in which the samplings were carried out (Fig. 6). The ANOSIM test demonstrates significant differences in species composition between the locations sampled in the Caatinga, Cerrado and Atlantic Forest (R = 0.662, P = 0.0001). Group 1 is formed by anuran assemblages sampled mainly in the Caatinga, where two main subdivisions can be highlighted, the first (1a) formed by localities along the middle Jequitinhonha River - MG and two localities in the Chapada Diamantina-BA, the latter hosting several endemic species (e.g., *Haddadusaramunha*, *Leptodactylusoreomantis*, *Rupiranacardosoi*) contributing to the unique anurofauna distinct from other sampled Caatinga locations (1b). Group 2 is formed by the anuran assemblages from the Cerrado.

Group 3 is comprised of anuran assemblages within the Atlantic Forest, and three main subdivisions can be highlighted. The first subdivision (3a) encompasses studies conducted in Dense Ombrophilous Forest in the extreme south of Bahia (e.g., RPPN Veracel, National Park of Descobrimento). It also includes transition areas influenced by Seasonal Forests located further inland, such as localities in the northeast of Minas Gerais (e.g., Complex Santana, Bandeira, and Limoeiro), and Serra Azul and Serra do Mandim in Bahia. The second subdivision (3b) formed by areas located in the south of the state of Bahia, primarily sampled in the Dense Ombrophilous Forest, and encompasses locations with the highest species richness in our analysis. Finally, the third subdivision (3c) encompasses transition areas between the Atlantic Forest and Caatinga (Brejo Novo, Morro do Mara), as well as Restinga areas (Conde) and Semideciduous Forest regions (RPPN Frei Caneca and Cruz das Almas).

### ﻿Taxonomic remarks

Some specimens encountered during field activities posed challenges for identification. Recently, Magalhães et al. (2020) revisited the *Leptodactyluslatrans* species group, employing multiple lines of evidence (DNA, acoustic, and morphological data), providing morphological diagnoses for the lineages encountered in their study. This facilitated the identification of two species from this group for the study area: *L.macrosternum* and *L.latrans*.

*Physalaemuserikae* was described in 2004 based on morphological and acoustic data, with the type locality in Guaratinga, Bahia (~ 100 km from the study area). This species closely resembles *P.kroyeri* but differs in having a shorter snout-vent length, a shorter advertisement call duration, and the presence of inguinal glands (Cruz and Pimenta 2004). Although our study area is close to the type locality, most specimens did not exhibit a visible inguinal gland, thus being considered as *P.kroyeri*. However, we encountered two specimens that exhibited an inguinal gland. For one of these, we recorded the advertisement call, which had a duration varying 0.37–0.46 s (MZUESC 15878). Recently, Hepp and Pombal (2020) reported that the advertisement call duration of *P.erikae* ranges from 0.478 to 0.566 s (*n* = 37 calls from four males), and for *P.kroyeri* it ranges from 0.673 to 0.759 s (*n* = 190 calls from eight males). Our data fall within the lower range of the reported variation for *P.erikae*. Braga et al. (2024) analyzed the vocalization of *P.kroyeri* from Cruz das Almas – Bahia (near the type locality) and found an advertisement call duration of 0.614 to 0.882 s (*n* = 83 calls from ten males), indicating a longer call duration in this species. However, we emphasize the need for a more comprehensive taxonomic revision of these two species, especially to increase the available data on the morphological and acoustic variation of *P.erikae*, particularly its acoustic parameters, especially those from its type locality, as well as the necessity of molecular sampling of topotypes of this species for inclusion in a phylogenetic approach.

Four species were not identified at the species level (*Adelophryne* sp. 2, *A.* sp. 8, *Ischnocnema* sp. [gr.parva] and *Pristimantis* sp. [grramagii]) and are considered candidate species that require further taxonomic investigation (Lourenço-De-Moraes et al. 2018; Trevisan et al. 2020; IR Dias, personal communication). Additionally, three other species (*Phyllodytesluteolus*, *Ischnocnemaverrucosa* and *Vitreoranaeurygnatha*) found in the area exhibit high intraspecific molecular diversity and may represent a similar case to the aforementioned (Blotto et al. 2021; IR Dias, personal communication; Zucchetti and Castroviejo-Fisher 2024).

## ﻿Discussion

During the present study, 46 amphibian species were recorded in Serra Azul and Serra do Mandim in southwestern Bahia. This diversity is likely to be even greater, as the species accumulation curves have not stabilized. Thus, in these forest remnants, isolated and immersed in a matrix dominated by pastures for livestock, approximately 25% of the species that occur in Bahia can be found (Dias et al. 2014b), showing the importance of maintaining and conserving the fragments of the region. The recorded richness ranks among the top ten highest ever found in the Northeast region of Brazil, with a high proportion of species endemic to the Atlantic Rainforest. However, it is important to note that the sampling effort among the different studied areas is highly unequal, making comparisons difficult. Localities where low sampling effort was employed, such as APA (Environmental Protection Area) Lagoa Encantada and Almada River (1 month) and RPPN Veracel (1 month), have a similar richness to places where sampling effort was much higher, such as the Michelin Ecological Reserve and RPPN Frei Caneca. It is likely that the anuran fauna in these areas where low sampling effort was employed is underestimated.

Among the species found, three are restricted to the Atlantic Forest of the state of Bahia: *Bahiusbilineatus*, Physalaemuscf.erikae, *Ololygonstrigilata* (Juncá and Pimenta 2004; Cruz and Pimenta 2004; Pimenta et al. 2007), and five others have a geographical distribution between southern Bahia and Espirito Santo or northeastern Minas Gerais: *Ischnocnemaverrucosa*, *Aplastodiscusweygoldti*, *Phyllodytesmaculosus*, *Dendrophryniscusproboscideus*, and *Leptodactylusviridis* (Caramaschi and Canedo 2006; Cruz et al. 2007; Moura et al. 2009; Lantyer-Silva et al. 2011; Silva et al. 2012).

Some of the species found occur mainly in open areas, such as *Rhinelladiptycha* and *Dermatonotusmuelleri* (Andrade and Carnaval 2004; Feio et al. 2006b), while others are considered typical of the Caatinga, such as *Leptodactylustroglodytes*, *Scinaxpachycrus*, and *Physalaemuscicada* (Feio and Caramaschi 1995; Rodrigues 2003; Peixoto and Arzabe 2004; Linares and Mello 2011). The encounter of species restricted to the Atlantic Forest, and others typical of the Caatinga, was expected since the study site is located in a transition area between these two regions. According to the similarity analysis, the composition of the anuran fauna of Serra Azul and Serra do Mandim is similar to other sampled areas in the region in ecotonal areas, which also share faunistic elements of these two morphoclimatic domains (Feio and Caramaschi 1995; Feio et al. 2006b).

Within the state of Bahia, some species found in the study area have records limited to fewer than three occurrence points, such as *Trachycephalusnigromaculatus*, and *Pseudisfusca* (Garda et al. 2010; Dias et al. 2010). *Pseudisfusca* (Fig. 3R), an aquatic species, is found in the states of Bahia, Minas Gerais, and Espírito Santo, Brazil (Garda et al. 2010). In Bahia, the species is known from two municipalities: Guaratinga and Teixeira de Freitas (Garda et al. 2010). The encounter of *P.fusca* in Serra Azul represents the third record of the species for the state of Bahia, marking an approximately 250 km straight-line increase in its geographical distribution from its type locality (Araçuaí – Minas Gerais). These new records contribute to a better understanding of the distribution patterns of these species in the region, especially for *P.fusca*, with this new occurrence point now representing the northern limit of the distribution of this species.

Amphibian inventories in southern Bahia have shown high species richness and endemism in the region (e.g., Silvano and Pimenta 2003; Camurugi et al. 2010; Rojas-Padilla et al. 2020; present study), which may be associated with the fact that the central region of the Atlantic Forest is estimated as a zone of climatic stability during the Quaternary glaciations, serving as a large refuge for amphibian species in the Atlantic Forest at the end of the Pleistocene, contributing to the maintenance of regional diversity (Carnaval and Moritz 2008; Carnaval et al. 2009).

Serra do Mandim and Serra Azul still have conserved forest fragments that form a complex network of streams that compose the Jequitinhonha river basins further south (IBGE 1997) and the Rio Pardo basin further north of the region (Pedreira 1999; Cetra et al. 2010). This great abundance of water bodies and the conservation of the study area contributed to the record of typical forest species (e.g., *Adelophryne* sp. 2, *A.* sp. 8, *Ischnocnemaverrucosa*, *Pristimantisvinhai*, and *Gastrothecapulchra*) and typical stream species (e.g., *Aplastodiscusweygoldti*, *Ololygonstrigilata*, and *Vitreoranaeurygnatha*). In addition, a high abundance of direct development species using leaf litter (e.g., *Pristimantisvinhai*, *P.* sp. [gr.ramagii] and *Haddadusbinotatus*), including species that are difficult to sample such as *Ischnocnemaverrucosa* and *Dendrophryniscusproboscideus*, was found, demonstrating that the sampled forest fragments still have adequate conditions for the maintenance of these populations in the region. Despite this, no protected area has been established to ensure the conservation of these elements of the region’s fauna. Actions are required to secure the preservation of these species.

Our results demonstrate that the remaining forest fragments in the region, although small and isolated, still support a high richness of amphibians with species restricted to the Atlantic Forest and Bahia, such as *Bahiusbilineatus* and *Ololygonstrigilata*, and others considered typical of the Caatinga, such as *Dermatonotusmuelleri*, *Leptodactylustroglodytes*, and *Physalaemuscicada*.

## ﻿Additional information

### Author ORCIDs

Mirco Solé https://orcid.org/0000-0001-7881-6227

Caio Vinicius de Mira-Mendes https://orcid.org/0000-0002-7707-6439

Iuri Ribeiro Dias https://orcid.org/0000-0002-2825-3494

### Data availability

All of the data that support the findings of this study are available in the main text.

## ﻿Appendix 1

List of vouchers deposited in the Museu de Zoologia da Universidade Estadual de Santa Cruz-MZUESC.

AMPHIBIANS

BRACHYCEPHALIDAE–*Ischnocnemaverrucosa*: MZUESC 15874, 15875, 15885, 15886, 15895, 15898, 15899. *Ischnocnema* sp. (gr.parva): MZUESC 15896. BUFONIDAE–*Dendrophryniscusproboscideus*: MZUESC 14688, 15126, 15127, 15128, 15798, 15803, 15859, 16532. *Rhinellacrucifer*: MZUESC 15031, 15148. *Rhinellagranulosa*: MZUESC 15030, 15055, 15504, 15505, 15506, 15850. *Rhinelladiptycha*: MZUESC 15503. CRAUGASTORIDAE–*Bahiusbilineatus*: MZUESC 15095, 15096, 15097, 15794, 15809, 15838, 15842, 15855, 15856, 15860, 15882, 15883. *Haddadusbinotatus*: MZUESC 15014, 15015, 15032, 15034, 15044, 15047, 15051, 15053, 15054, 15057, 15058, 15104, 15105, 15106, 15107, 15131, 15142, 15465, 15471, 15472, 15473, 15475, 15476, 15478, 15479, 15482, 15526, 15622, 15623, 15634, 15635, 15643, 15644, 15646, 15650, 15651, 15656, 15659, 15660, 15778, 15802. *Pristimantisvinhai*: MZUESC 15000, 15098–15103, 15109–15112, 15114, 15115, 15120, 15121, 15132, 15133, 15140, 15141, 15143, 15144, 15464, 15481, 15502, 15640, 15642, 15657, 15663, 15664, 15667, 15780, 15785, 15786, 15797, 15799–15801, 15806–15808, 15813–15815, 15837, 15840, 15841, 15843, 15853, 15854, 15857, 15858, 15861, 15879, 15897, 15889. *Pristimantis* sp. (gr.ramagii): MZUESC 15033, 15474, 15641, 15658, 15662, 15665, 15783, 15849, 15851, 15852, 16523. CENTROLENIDAE–*Vitreoranaeurygnatha*: MZUESC 14691, 14698, 14699, 14700, 14701, 15810–15812. CYCLORAMPHIDAE–*Thoropamiliaris*: MZUESC 14674, 15130, 15477, 15782, 15789, 15845, 15846, 15872, 15873, 15880, 15881. ELEUTHERODACTYLIDAE–*Adelophryne* sp.8: MZUESC 15035, 15036, 15039, 15041, 15043, 15048, 15050, 15052, 15116, 15462, 15466, 15480, 15528–15531, 15625–15627, 15631, 15633, 15637–15639, 15647, 15652, 15661, 15796, 15830, 15834, 15835, 15839, 15862, 16522, 16530. *Adelophryne* sp.2: MZUESC 15024, 15037, 15038, 15040, 15042, 15045, 15046, 15049, 15090, 15091, 15094, 15113, 15118, 15119, 15123–15125, 15457–15460, 15467–15470, 15524, 15525, 15527, 15533, 15624, 15628, 15629, 15632, 15636, 15645, 15648, 15649, 15655, 15795, 15831, 15832, 15836. HYLIDAE–*Aplastodiscusweygoldti*: MZUESC 14690, 15787, 15788, 15844, 15887. *Boanacrepitans*: MZUESC 14673, 14675, 14998, 14999, 15002, 15145, 15486. *Boanaexastis*: MZUESC 15108. *Boanafaber*: MZUESC 14676, 15066, 15068, 15147, 15666, 15884. *Dendropsophusbranneri*: MZUESC 14683, 14684, 15022, 15023, 15509, 15510, 15512, 15818, 15824. *Dendropsophuselegans*: MZUESC 14678, 14679, 15018, 15019, 15020, 15021, 15138, 15507, 15508, 15820. *Dendropsophusoliveirai*: MZUESC 14686, 15010, 15011, 15487–15501, 15511, 15791, 15792, 15823, 15825. *Ololygonstrigilata*: MZUESC 15001. *Phyllodytesluteolus*: MZUESC 17501, 17502. *Pithecopusnordestinus*: MZUESC 14680–14682, 14685, 14695, 14997, 15136, 15137, 15826, 15827, 16527. *Pseudisfusca*: MZUESC 16528. *Scinaxeurydice*: MZUESC 14672, 14693, 15876, 16531. *Scinaxpachycrus*: MZUESC 15790, 16525. *Scinaxx-signatus*: MZUESC 14692, 14694, 15027, 15139, 15892, 15894, 17503. *Sphaenorhynchusprasinus*: MZUESC 14677, 15004–15009, 15016, 15017, 15135, 15484, 15485, 15793, 15821, 15822. *Trachycephalusnigromaculatus*: MZUESC 15063, 15064, 15065, 15483, 15888. LEPTODACTYLIDAE–Leptodactyluscf.mystaceus: MZUESC 14696. *Leptodactylusfuscus*: MZUESC 15012, 15067, 15513–15516. *Leptodactyluslatrans*: MZUESC 15871, 15893. *Leptodactylusmacrosternum*: MZUESC 15146, 16524. *Leptodactylusmystacinus*: MZUESC 16529, 16533. *Leptodactylustroglodytes*: MZUESC 15003, 15028, 15029. *Leptodactylusviridis*: MZUESC 15848, 15869, 15870. *Physalaemuscicada*: MZUESC 15059–15062. Physalaemuscf.erikae: MZUESC 15013, 15878. *Physalaemuskroyeri*: MZUESC 15517, 15518, 15521, 15523, 15784, 15819, 15877. MICROHYLIDAE – *Dermatonotusmuelleri*: MZUESC 15069, 15070. ODONTOPHRYNIDAE–*Proceratophrysschirchi*: MZUESC 14689, 14702–14705, 15071, 15072, 15122, 15134, 15779, 15781, 15804, 15816, 15817, 15833, 15847, 15863–15868. PIPIDAE–*Pipacarvalhoi*: MZUESC 15129. SIPHONOPIDAE–*Siphonopsannulatus*: MZUESC 15900.
